# Menstrual food restrictions and taboos: A qualitative study on rural, resettlement and urban indigenous Temiar of Malaysia

**DOI:** 10.1371/journal.pone.0279629

**Published:** 2022-12-27

**Authors:** Sharifah Zahhura Syed Abdullah

**Affiliations:** Unit for Research on Women and Gender, School of Social Sciences, Universiti Sains Malaysia, George Town, Malaysia; UCSI University, MALAYSIA

## Abstract

Menstruation is arguably the first stage in a woman’s reproductive cycle. Among the Temiar, as in many other traditional societies, menstruation represents a time during which a woman is considered to be vulnerable or polluted and there may be food or behavior avoidances and restrictions. The Temiar is one of the eighteen indigenous sub-ethnic groups in Peninsular Malaysia. The objective of this study was to examine the food restrictions and taboos imposed on menstruating Temiar women. A total of 38 participants from four different locations took part in five focus group discussions which represents different lifestyle experiences of the Temiar sub-ethnic group. The findings unfolds many practices: foods to be avoided and spirit in the landscape in order to protect the menstruating woman; isolating the menstruating woman in order to protect the community; consequences of not observing the menstruation food taboos and maintenance of the menstrual taboos. The menstruating women in all locations were prohibited from consuming salt, cooking oils, wild or domesticated animals, and Monosodium glutamate to protect themselves from the excessive flow of menstrual blood and future ill-health. They must eat separately from others because they are deemed polluted and dangerous to the community. The study concludes that the taboos directed towards the menstruating women often do have a caring and protective intention. Menstrual restrictions function not only to protect the menstruating women and the community but also to keep intact the symbolic boundary between human and the non-human world from which disease and weakness comes.

## Introduction

Menstruation is a physiological process that is experienced almost universally across cultures from the ages of menarche to menopause [1]. The term “menstruation” derives from the Latin word “menses”, which means moon, and refers to the lunar month, that lasts about 28 days [2]. In many cultures around the world, menstruation has always been surrounded by taboos, myths and restrictions intended at protecting not only the menstrual girls and women but also the community. Some of these are beneficial, but others have the potential to be harmful [3].

There have been numerous studies on traditional and religious beliefs relating to menstruation and the dangers of female pollution. In the cultural tradition of many societies, the menstruating female body is regarded as polluted and polluting [4]. Menstruating women do not only have to practice taboos imposed on them by their cultures, they also have to obey restrictions placed on them by their religions. According to Guterman, Mehta and Gibbs [5], many religions still have enduring beliefs and notions regarding menstruation and that there are similarities in beliefs and taboos about menstruation among the major religions. Menstruating women are considered ritually unclean in Judaism, dangerous in Christianity, impure and polluted in Hinduism, and polluted in Buddhism. Islam does not view a menstruating woman as having any sort of “contagious uncleanness”. She is neither “cursed” nor “untouchable” [6]. Certain taboos and social restrictions are directed at menstruating women in all of these religions [5]. For example, Pedersen [7] in her study among Hindu Balinese in Indonesia, found that, “the menstruating woman and her blood are explicitly considered to be polluted”. Accordingly, Balinese women are sometimes isolated from other people due to their “impure” status [8]. Among the Muslims, a menstruating woman is not dirty; rather she is ritually impure for the duration of her menstruation. Ritual impurity is a condition that prevents a menstruating woman from engaging in specific acts of worship such as daily prayers and fasting until the condition is removed by purification bath [9]. The origins of menstrual taboos may well be rooted in ancient animist beliefs. Thomas [10] explains in her study of menstruation taboos in both Western and non-Western cultures that in ancient times, taboos were created around elements such as blood pollution produced by the female body.

Later, in the early modern period, some societies believed that menstruating women are unclean and dirty [11,12] and untouchable [2,13]. Menstruation was categorised as “dirty” or “dangerous” in order to establish a social structure and justify behaviour and to create divisions between men and women [10] and “to preserve male dominance by stigmatizing women and their sexuality as a supernatural threat to men and the social and cultural order men represent and control” [14]. Taboos and restrictions set the menstruating women apart from the rest of their own society [11,12] in order to protect the rest of the community [15]. However, Lhamo [16], in her article on female pollution in Taiwanese women, reported that menstruating women were considered vulnerable and an easy target for spirits, demons, or ghosts. Where as in Tanzania, some girls said that they were taught not to throw discarded menstrual materials out in the open because they could be utilised in witchcraft, resulting in death or infertility [12].

The Temiar, a distinctive ethnic group among the Orang Asli or indigenous people in Peninsular Malaysia, have their own distinctive menstrual taboos and restrictions. These traditional taboos and restrictions are deeply rooted in their culture, customs, values and beliefs system. As an animistic people, they believed that all entities, human and non-human, seen and unseen, were embodied with a soul or spirit. Temiar people had a rich spiritual life in which these many different entities were recognised and reconciled [17]. In recent years, processes of relocation and migration have given fewer opportunities for them to maintain their traditional knowledge of menstrual taboos and restrictions. Considering the limited research so far available on menstruation especially among the Temiar, the study attempts to redress the gap in the literature through gaining an understanding of the menstrual taboos and other restrictions that Temiar women follow during the menstruation period and the rationale underpinning such practices.

## Methods

### Research locations

A qualitative approach was taken in this study to compare the cultural rules and patterns among Temiar living in contrasting social and ecological environments. Four different locations in this research represent different lifestyle experiences and cultural practices of the Orang Asli Temiar.The locations ranged from the most traditional locations, Pos Simpor and Pos Tohoi in Kelantan, representing the experiences of Temiar who live in or close to the forest, to Rancangan Pengumpulan Semula Orang Asli(Orang Asli (Regroupment Scheme) or RPSOA in Kuala Betis, Kelantan, a resettlement area, representing a transition from forest to town living, and the urban area, Batu 12 in Gombak, Selangor, representing the Temiar who now live in town.

### Participants

In each fieldwork location, participants were identified using a snowball approach. This sampling technique was chosen because as indigenous people, the Temiar as study populations would be best approached by people they knew in the first instance rather than by an outsider. First, the official key informant was contacted, primarily the Tok Batin, or a local JAKOA (Department of Orang Asli Development) staff member. The researcher alerted that person to the research and asked for suggestions for potential participants. Each was a local person who was active in, or had connections to, the Temiar community in that location. They helped to locate and select potential participants since they knew how to gain access to the population, how best to approach people, and any possible obstacles that might occur. A total of 38 participants consisting of 20 females and 18 males, aged 19 to 67 years old took part in five focus group discussions. One group (five male participants) in Pos Simpor; one group (ten participants including three females and seven males) in Pos Tohoi; two groups (one group consisting of nine female participants and another group consisting of six male participants) in RPSOA in Kuala Betis; and one group (eight female participants) in Batu 12, Gombak (S1 Table). The sample size of five focus groups were enough to identify most of the prevalent themes within the data set. For purposes of a comparative study, each focus group represented a location in which different lifestyle experiences of the Orang Asli Temiar sub-ethnic group indicated that something different might be said about food taboos and restrictions during menstruation.

#### Data collection

Data collection took place between January and June 2008. The qualitative method used in this study was focus group discussions (FGDs). FGDs increased the range of types of participants and covered the dimensions of diversity that seemed most relevant to the research topic [18]. FGDs consisted of the selected key informants (Tok Batin or head village, health officers and midwives), and older and young cohorts of women and men. Participants were provided a detailed explanation of the purpose and scope of the study, as well as an informed consent form. Written consent was taken from each participant before focus group discussion. The researcher’s presence as facilitator during focus group discussion, which is often unavoidable in qualitative research, may have somehow affected the participants’ responses. Thus, all focus group discussions took place in a familiar setting in order to motivate participants and to create a comfortable environment. Each session was audio-taped and video-recorded to allow the facilitator to focus on group responses and non-verbal behaviour, and for later transcription.

### Ethical considerations

The questions for focus groups were developed to ensure consistency in responses according to project themes while allowing for flexibility of later interpretations. The focus groups were conducted ‘in the field’ in familiar surroundings for participants to ensure cultural comfort and the best possible responses. The research procedures were fully approved by the Human Research Ethics Committee of the University of Newcastle, Australia (approval number H-592-0907).

### Data analysis

Focus group discussions were audio-taped and video-recorded (with permission) and these tapes were then transcribed from oral language to written language. Observation notes were analysed for patterns, themes and categories. All the transcripts were coded and categorised as either a main category or sub category and then ‘thematised’ using the software package for handling qualitative data, NVivo. Each participant names have been replaced with pseudonym to protect confidentiality. All data containing identifying information such as names is stored securely.

## Results and discussion

Spielmann [19] concludes that avoidances are usually placed on women during critical times in their reproductive cycle, when their energy requirements are highest [20] for example during pregnancy, the postpartum period and menstruation. Among the Temiar, as in many other traditional societies, menstruation represents a time during which a woman is considered to be vulnerable/polluted and there may be food or behaviour avoidances and restrictions. The following Table 1 summarises the data.

**Table 1:** Restricted food items and other avoidances imposed on women during menstruation, supposed effect of these restrictions and avoidances, who is supposed to be protected, and research locations involved.

Restricted food items and other avoidances	Research locations	Supposed effect if violated	Supposed to protect
1. Animals (game or domesticated)	All locations	• Excessive flow of menstrual blood • Future ill-health	Menstruating woman
2. Salt	All locations	• Excessive flow of menstrual blood • Future ill-health	Menstruating woman
3. Cooking oil	All locations	• Excessive flow of menstrual blood • Future ill-health	Menstruating woman
4. Monosodium Glutamate	All locations	• Excessive flow of menstrual blood • Future ill-health	Menstruating woman
5. Glutinous rice	Batu 12, Gombak	• Cold and shaking	Menstruating woman
6. Eat separately	All locations	• Deemed as polluted and dangerous	Other people, community
7. Wash at the river (non-food)	RPSOA Kuala Betis	• Easy target for the spirits, demons or ghost • Menstrual blood will keep flowing out	Menstruating woman

### Foods to be avoided: Protecting the woman

Participants in all locations indicated that menstruating women must refrain from eating certain kind of foods. They related their claims to consequences of bad health or bad luck. They explained that menstruating women were only allowed to eat rice or cassava root without any other side dishes. As indicated in the table, they were forbidden from eating meat of game or domesticated animals, salt, Monosodium glutamate (MSG), and cooking oil. Taboos against these food items served to protect the menstruating women from powers supernaturally harmful to them in their vulnerable state, and also to protect other people. Only FGD participants from Batu 12, Gombak, the urbanised location, mentioned avoiding glutinous rice. Here it was indicated that if this restriction were violated by a menstruating woman, she would feel cold and start to shake, “If we eat glutinous rice during the menstruation period, we will start shaking due to feeling cold” (Semah, female, 63 years old).

Participants from other locations did not mention this restriction. Either they forgot about it or, even more likely, glutinous rice was difficult to obtain in their locations and thus it did not immediately come to mind as one of the menstruation taboos. In general, it seemed food restrictions were followed by the menstruating woman for one week or until the bleeding stopped.

Menstrual food taboos and avoidances in all culture followed sets of rules to maintain an equal and balanced harmony between all entities and to prevent any misfortune or calamity from happening. In some parts of India, some strict dietary restrictions are also followed during menstruation such as sour food like curd, tamarind, and pickles are usually avoided by menstruating girls [21]. It is believed that such foods will disturb or stop the menstrual flow [22]. A study by Mohamed and her colleagues [11] found that when it comes to menstruation, some female participants believe that fish, meat, and other proteins should be avoided since they make the menstrual flow heavier or more ’smelly.’ Both studies emphasized that dietary restrictions were followed in order to protect the menstruating women. However in Nepal, the restriction placed was not to protect the girls or women, they were restricted from eating dairy products because they believed that it would cease the lactation of the cattle [13].

### Isolating the menstruating woman: Protecting the community

There was widespread agreement that a menstruating woman must eat alone, from a special set of dishes, a practice that manages the risk of her polluting others. The general consensus in all locations was that a menstruating woman must not share her meal with other family members or people. The following statements show the strength and unity of this belief:

She eats separately, cannot mix with others. (Salim, male, 37 years old, Pos Simpor)

The women are not allowed to eat together. (Zakaria, male, 39 years old, Pos Tohoi)

If the woman has her monthly period, ha… she cannot eat together with her mother and father, she must eat alone. (Kinah, female, 36 years old, RPSOA Kuala Betis)

But she must eat separately; she cannot eat with her mother or siblings. (Rohani, female, 33 years old, Batu 12, Gombak)

Temiar in all locations, even the most urbanised, still restrict the menstruating woman to eating alone, from a separate set of dishes. During earlier times, a similar belief exist among the Bhargava Brahmins girls during menarche where they used to eat in separate utensils [21]. While in some very remote parts of Solomon Island and Papua New Guinea, women and girls stay in a separate women’s house (‘haus meri’) every month for the duration of their menstrual period and eat alone [11]. Where as in Nepal, menstrual exile or Chhaupadi is a centuries-old cultural and religious practice in which Hindu women and girls are excluded from society and family life and banished to makeshift hut or livestock shed while they are menstruating [13]. They are frequently separated from communal spaces and events in order to contain pollution [4]. For the Temiar, the restriction is obviously aimed at protection of the community rather than the individual woman. It seems that this is a particularly strong belief that has not been much affected so far by the influence of urbanised modernity. Similarly, the women in Papua New Guinea and Solomon Island believed that exposure to menstruating women or menstrual blood can cause bad luck for men and boys, negatively impacting their health and physical strength and ability to hunt, fish and play sports [11].

### Consequences of not observing the menstruation food taboos

When asked why the woman should refrain from eating certain foods during the menstrual period, participants from RPSOA Kuala Betis, the resettlement area, answered that it was to prevent excessive flow of menstrual blood: “We fear that if all these restrictions are ignored, it will cause a lot of blood” (Fatimah, female, 34 years old). However, study participants from Pos Tohoi, a traditional, remote location and Batu 12, Gombak, the most modern, urban location, put more emphasis on the idea that food restrictions prevented long-term negative residual effects on the woman’s body during this vulnerable/polluting period, for example:

Ha… for us, she eats no salt, similar to restrictions after childbirth. Because if she disobeys and eats it she will get sick or get a cough or other sicknesses of that kind. (Penghulu, male, 67 years old, Pos Tohoi)

Ha… if when we are young we ignore the avoidances, when we get older, we will get asthma, a thin body, and be diseased. (Semah, female, 63 years old, Batu 12, Gombak)

Menstrual restrictions function not only to protect the menstruating women and the community but also to keep intact the symbolic boundary between human and the non-human world from which disease and weakness comes. For example, menstruating women and girls in Nepal adhere to the Chhaupadi which was primarily motivated by family custom because they believed that failing to do so would bring misfortune for example death to the family [13]. Correspondingly, a study in far-west Nepal found that the participants were afraid of numerous negative consequences that were health-related as a result of not following menstrual traditions such as pain and sickness, health problems for their mothers, headaches, shivering, leg pain, teeth pain, and/or stomach aches [4].

### Spirits in the landscape: Protecting the menstruating woman

Only participants from the resettlement area RPSOA Kuala Betis mentioned that during the menstrual period women were not supposed to go to, or enter, the river for any reason, for example, “She can wash using the piped water but she cannot go to the river” (Kinah, female, 36 years old). RPSOA Kuala Betis is situated near a point of two merging rivers, Sungai Nenggiri and Sungai Galas, which flow into the Sungai Kelantan before reaching the sea at Kota Bharu.

In the same focus group, another female participant claimed that if the menstruating women went to the river too soon, this would increase the menstrual flow again. She explained:

We are afraid of it coming back… in the afternoon the period will be there again, it will come back. (Timah, female, 40 years old)

Some of the findings from this study do not precisely match previous findings on Temiar menstrual taboos. For example, in her earlier study of the Temiar, Jennings [23] found that during menstruation, Temiar girls observed not only the food restrictions described above, but specifically avoided contact with men because menstrual blood was regarded as dangerous to all men. Specific avoidance of men was not mentioned in the focus groups that were conducted in this study. Statements about isolation appeared to include men, women and family members. However, the taboo on river contact did correspond. Jennings [23] found there were various evil, supernatural creatures believed by the Temiar to inhabit river banks and river mouths: they can attack people, and invade their bodies, and even worse, their souls. Based on this study’s findings and Jennings’ findings, it is inferred that the logic here is that menstruating women should avoid the river for their own protection, because women are vulnerable at this time and will be an easy target for evil spirits to invade their bodies.

This taboo can be identified, using an extension of Aunger’s [24] taxonomy, as a spiritual belief. If a Temiar person is attacked and possessed by a demon, ghost, or spirit, the condition will need to be treated by the *Halaq* who will perform an exorcism ritual. The example of the river taboo indicates that the very fact of menstruation (bleeding) is believed to attract negative supernatural forces. These evil creatures are implicitly threatening not just to the menstruating woman herself, as indicated by research findings on indigenous folk beliefs elsewhere in East Asia. For example, Lhamo [16], in her article on fears about female pollution in Taiwanese society, found the following belief: because the menstruating woman loses blood, this attracts hungry ghosts who feed on blood. When the hungry ghosts come, they threaten other people around the menstruating woman too. This is addressed by separation practices. The same deep logic related to ancient animist beliefs may also apply in the case of the Temiar. The “separation” of women from the river during the menstruating period functions to avoid attracting the malevolent spirit’s attention. Austronesian cultural systems treat menstrual blood as spiritually unclean and dangerous because it violates culturally defined cosmological boundaries. The rules that are expressed in the telling of proverb-like sayings for example, “Her spirit will be taken from her by the river water” (Kinah, RPSOA Kuala Betis) preserve a boundary between dangerous spirit powers and the human realm.

Restriction on bathing was found in a study conducted by Kumar and Srivastava [21] among adolescent girls in Ranchi. It was discovered that girls in slum areas believe that bathing during menstruation will increase the flow of menstrual blood, whereas Muslim girls believe that bathing during this period will increase intricacies during pregnancy. Similarly the restriction placed was to protect the individual women but has nothing to do with attack by malevolent spirits.

### Maintenance of menstrual taboos

When FGD participants at all four locations were asked whether they still followed the restrictions and avoidances placed upon the menstruating woman, most responded that Temiar women generally adhere to certain menstrual taboos as a regular and accepted part of their lives. Menstruation taboos are viewed auspiciously and actively followed by Temiar women in the remote areas of Pos Simpor, Pos Tohoi. They are also followed by some in the resettlement area of RPSOA Kuala Betis. Unsurprisingly though, most female participants from the most urbanised location of Batu 12, Gombak said that they were no longer obeying the traditional menstrual avoidances. They felt their children and grandchildren should be free to choose whether to obey the menstrual taboos or not. One female participant from Batu 12, Gombak said, “Now we are free, that is why we cannot forbid our children, our grandchildren… he… he… he…”(laughing) (Semah, 63 years old). A similar belief exist among women and girls in Papua New Guinea who described that these traditional restrictions on cooking and housework were becoming less common and were often not adhered to, particularly in urban areas [11].

Yet according to one Temiar informant who lives and works in a Malaysian city other than Batu 12, Gombak, she still obeys one traditional restriction during her menstrual period. She cooks a meal, and then separates an unsalted portion for herself. When inquired why she should refrain from salt, she answered that it was passed to her from her mother, who had received the taboo from her mother, and so on. The informant had been told by her mother not to eat food containing salt when she was menstruating. This finding matches the convention in indigenous societies that parents should transmit core beliefs to their offspring of the same gender. The mother of this informant had obviously remembered her mother’s advice on avoidance of salt throughout her life and at the appropriate point in her own daughter’s life had gone through the same instructional process with her. When asked what this informant ate during office hours while menstruating, she said that she ate bread or something similar. It is notable that even though she grew up in a modern urban environment, and did not have much knowledge about consequences if the restrictions were violated, she was still observing this menstrual restriction. This example shows how traditional Temiar precautions and avoidances descended from the ancestors can still serve as a guide for regulating the menstruating woman’s behaviour, even in a very modern, urban context. Similarly, the taboos practised among adolescents girls in Ranchi are still prevalent, however they are seen as threats and need to be considered seriously by the professionals in the health sector [21].

### Avoiding cold foods during menstruation

In other relevant studies in Southeast Asia, it has been found that certain food taboos were derived from humoral notions of the body in terms of the categories ‘hot’ and ‘cold’ [25,26]. However, there was little or no standard categorisation or classification of food taboos in those terms offered by the Temiar FGD participants. None of the participants mentioned restricted food items specifically under ‘cold’ or ‘hot’ or ‘neutral’ categories. Instead, they talked about the supposed effects of particular foods on the baby, mother, father, or other people, and these effects only rarely included making the person “hot” or “cold”. The most common general effect was *sawan* or illness.

There was only one instance where “coldness” was claimed: that of the taboo against a menstruating woman consuming glutinous rice because it would make her feel cold. Glutinous rice might therefore be considered as a “cold” food based on the humoral system. My findings support Laderman’s claim that even though hot-cold opposition is dominant, the Orang Asli do not employ a humoral system in their medical theories. Furthermore, the claim about glutinous rice was only made at Batu 12, Gombak, where this food stuff is available, and where there is much contact with other belief systems, cultures and religions. It is therefore difficult to define it as a distinctive Temiar belief.

## Limitations and study forward

Firstly, the sampling strategy resulted in a group of adult women and men who expressed adult opinions and points of view. With only a small number of youth involved in the focus group discussions, their voices were seldom heard because they were overpowered by the elders and perhaps thought their opinion and knowledge might be wrong. A wider sample of youth and a separate focus group discussion with them may have generated more data to shed light on the opinions and knowledge of young people about their menstrual culture and customs.

The Temiar are found in three states of Peninsular Malaysia: Perak, Kelantan and Selangor. Nevertheless this research was conducted only in two states, Kelantan and Selangor. Since the Temiar who live in Perak come from the same sub-ethnic group, it would seem they had a similar culture, language and lifestyle to the Temiar in Kelantan and Selangor. However, there might be differences in other aspects, such as customs. Hence, the findings cannot be generalizable for the entire Orang Asli Temiar population in Peninsular Malaysia especially the Temiar living in Perak and the addition of a research location at Perak would have enriched the study.

## Conclusion

The taboos directed towards the menstruating women often do have a caring and protective intention. Both kinds of protection are evident in the taboos and restrictions that govern the menstruating Temiar woman. Menstruation taboos are viewed auspiciously and actively followed by Temiar women in the remote areas. They are also followed by some in the resettlement area. Unsurprisingly though, most female participants from the most urbanized location were no longer obeying the traditional menstrual avoidance. Menstrual restrictions function not only to protect the menstruating women and the community but also to keep intact the symbolic boundary between human and the non-human world from which disease and weakness comes.

## Supporting information

S1 Table Study flow chart. (DOC)
