# Supplementary material for: Menstrual food restrictions and taboos: A qualitative study on rural, resettlement and urban indigenous Temiar of Malaysia
Source: PLoS One. 2022 Dec 27;17(12):e0279629. doi: 10.1371/journal.pone.0279629 (PMC9794040; doi:10.1371/journal.pone.0279629)
Supplement: S1 Table — (DOC) [file pone.0279629.s001.doc]

**S1 Table: Study flow chart**

**Identification of Research Locations and Study Populations**

**Human Research Ethics Approval**

**Department of Orang Asli Development, Malaysia (JAKOA) Approval**

**Focus group discussions** (n=38)

Pos Simpor - one group: n=5 (male participants)

Pos Tohoi - one group: n=10 (3 female and 7 male participants)

RPSOA Kuala Betis - two groups:

group 1: n=9 (female participants)

group 2: n=6 (male participants)

Batu 12, Gombak - one group: n=8 (female participants)

**Data analysis using NVivo**

**Interpreting data and Report writing**
